# DEK Is a Potential Biomarker Associated with Malignant Phenotype in Gastric Cancer Tissues and Plasma

**DOI:** 10.3390/ijms20225689

**Published:** 2019-11-13

**Authors:** Kam-Fai Lee, Ming-Ming Tsai, Chung-Ying Tsai, Chung-Guei Huang, Yu-Hsiang Ou, Ching-Chuan Hsieh, Hsi-Lung Hsieh, Chia-Siu Wang, Kwang-Huei Lin

**Affiliations:** 1 Department of Pathology, Chang Gung Memorial Hospital, Chiayi 613, Taiwan; lkf2002@cgmh.org.tw; 2 Department of Nursing, Division of Basic Medical Sciences, Chang-Gung University of Science and Technology, Taoyuan 333, Taiwan; mmtsai@mail.cgust.edu.tw (M.-M.T.); hlhsieh@gw.cgust.edu.tw (H.-L.H.); 3 Research Center for Chinese Herbal Medicine, College of Human Ecology, Chang Gung University of Science and Technology, Taoyuan 333, Taiwan; 4 Department of General Surgery, Chang Gung Memorial Hospital, Chia-yi 613, Taiwan; p12155@cgmh.org.tw; 5 Department of Biochemistry, College of Medicine, Chang-Gung University, Taoyuan 333, Taiwan; cytsai0616@cgmh.org.tw (C.-Y.T.); planet7668@hotmail.com (Y.-H.O.); 6 Kidney Research Center and Department of Nephrology, Chang Gung Memorial Hospital, Taoyuan 333, Taiwan; 7 Department of Medical Biotechnology and Laboratory Science, and Graduate Institute of Biomedical Science, Chang Gung University, Taoyuan 333, Taiwan; joyce@adm.cgmh.org.tw; 8 Department of Laboratory Medicine, Chang Gung Memorial Hospital, Linkou, Taoyuan 333, Taiwan; 9 Department of Neurology, Chang Gung Memorial Hospital, Taoyuan 333, Taiwan; 10 Liver Research Center, Chang Gung Memorial Hospital, Linkou, Taoyuan 333, Taiwan

**Keywords:** DEK, biomarker, gastric cancer, metastasis, prognosis, diagnosis

## Abstract

Gastric cancer (GC) is the second most widespread cause of cancer-related mortality worldwide. The discovery of novel biomarkers of oncoproteins can facilitate the development of therapeutic strategies for GC treatment. In this study, we identified novel biomarkers by integrating isobaric tags for relative and absolute quantitation (iTRAQ), a human plasma proteome database, and public Oncomine datasets to search for aberrantly expressed oncogene-associated proteins in GC tissues and plasma. One of the most significantly upregulated biomarkers, DEK, was selected and its expression validated. Our immunohistochemistry (IHC) (*n* = 92) and quantitative real-time polymerase chain reaction (qRT-PCR) (*n* = 72) analyses disclosed a marked increase in DEK expression in tumor tissue, compared with paired nontumor mucosa. Importantly, significantly higher preoperative plasma DEK levels were detected in GC patients than in healthy controls via enzyme-linked immunosorbent assay (ELISA). In clinicopathological analysis, higher expression of DEK in both tissue and plasma was significantly associated with advanced stage and poorer survival outcomes of GC patients. Data from receiver operating characteristic (ROC) curve analysis disclosed a better diagnostic accuracy of plasma DEK than carcinoembryonic antigen (CEA), carbohydrate antigen 19.9 (CA 19.9), and C-reactive protein (CRP), highlighting its potential as an effective plasma biomarker for GC. Plasma DEK is also more sensitive in tumor detection than the other three biomarkers. Knockdown of DEK resulted in inhibition of GC cell migration via a mechanism involving modulation of matrix metalloproteinase MMP-2/MMP-9 level and vice versa. Our results collectively support plasma DEK as a useful biomarker for making diagnosis and prognosis of GC patients.

## 1. Introduction

Gastric cancer (GC) is the second most common cause of cancer-related death on a global scale [1]. The Department of Health of Taiwan reported GC as the seventh main cause of cancer-associated mortality in 2018 [2]. Despite recent improvements in diagnostic and therapeutic technologies, a large proportion of GC patients have poor survival, primarily because initial diagnosis is not made at the early stages. The recent advent of serum biomarkers offers a non-invasive and convenient method for diagnosis and monitoring of GC [3]. However, the currently available GC biomarkers, such as carcinoembryonic antigen (CEA) and carbohydrate antigen 19.9 (CA 19.9), have limited diagnostic sensitivity and specificity [4]. Previous studies by our group showed that higher serum C-reactive protein (CRP) levels are associated with advanced GC stages and poorer outcomes, suggesting that preoperative CRP may serve as a potential prognostic biomarker [5]. Suitable biomarkers with improved efficacy in monitoring disease progression should effectively facilitate earlier diagnosis and treatment of GC [6].

Proteomic approaches are powerful tools for biomarker identification in tissue specimens but not blood specimens of malignant tumors. Biomarkers for tumor screening are more easily detected in blood samples from patients, as proteins are secreted by or shed from tumor cells into the blood. The human plasma proteome database has been reported to assist as a reference platform for biomarker discovery [7]. However, problems remain regarding the depth to which these databases can be mined and the efficiency with which researchers can select useful candidates from the identified proteins. Here, we have presented a strategy to identify potential blood biomarkers overexpressed in GC tissue and secreted into peripheral blood. Oncomine (genomic) datasets (https://www.oncomine.org) were integrated with GC cDNA microarrays, and iTRAQ (proteomic) datasets as well as a human plasma proteome database (http://www.plasmaproteomedatabase.org/) to determine potential factors aberrantly regulated and secreted or released from GC tissues. Among the novel candidate proteins identified in our GC specimens, DEK was selected for further validation.

DEK is a well-known proto-oncogene found in a range of nuclear proteins involved in chromatin remodeling, transcriptional repression or activation, DNA damage repair, cell proliferation, and suppression of apoptotic pathways [8]. DEK overexpression has been documented in various human malignant tissues, including GC, pancreatic ductal adenocarcinoma, oral squamous cell carcinoma (OSCC), hepatocellular carcinoma (HCC), chronic lymphocytic leukemia (CLL), lung cancer, cervical cancer, breast cancer, melanoma, head-and-neck cancer, bladder cancer, retinoblastoma, T-cell large granular lymphocytic leukemia, colon cancer, prostate cancer, acute myeloid leukemia (AML), and malignant glioma melanoma [8,9,10,11,12,13,14,15,16,17,18,19,20,21]. Moreover, DEK has been detected extracellularly in the urine of patients with bladder cancer and shown to be released by activated macrophages in the hemopoietic system [11]. HepG2 cells are reported to secrete DEK peptide in conditioned media [22,23]. DEK can also be recognized by specific antibodies in autoimmune disease [24,25] or taken up as a functional exogenous protein by nearby cells, in turn stimulating chronic inflammation and inducing more proinflammatory factors that generate progressive tumor microenvironments [26]. In this study, the potential role of DEK as a biomarker in both blood and tissue specimens of GC patients was investigated.

## 2. Results

### 2.1. Identification and Validation Studies for DEK, a Potential Biomarker for GC

**Figure 1:**
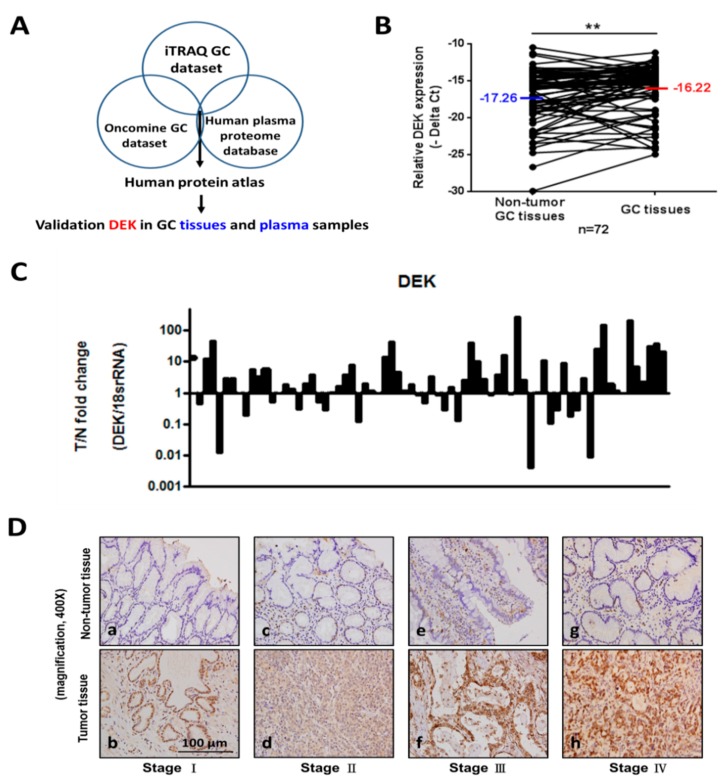
Identification and validation studies for DEK, a potential marker for gastric cancer (GC). (**A**) Identification of potential GC tissue/plasma biomarkers based on combined data from the iTRAQ GC dataset, Oncomine GC dataset, and human plasma proteome database. The strategy comprises genomic and proteomic profiling and subsequent validation in clinical specimens. (**B**) Relative expression levels of DEK in paired GC and adjacent normal tissues (*n* = 72) determined via quantitative real-time polymerase chain reaction (qRT-PCR) and GAPDH normalization (*p* = 0.0059) using paired sample *t*-tests. Error bars indicate standard deviations. (**C**) Distribution curves are shown as histograms for the same data. The DEK level was upregulated in GC tumors (T) relative to paired normal tissues (*n*). (**D**) Immunohistochemistry (IHC) staining of GC tissues for DEK. Representative staining results from four pairs of GC (lower panel) and adjacent normal tissues (upper panel). Differentially expressed DEK levels in tumor cells are depicted at the top of the panel. The Mann–Whitney *U* test was used for comparison between the two groups (* *p* < 0.01, ** *p* < 0.05, *** *p* < 0.001).

To accelerate the discovery of potential GC biomarkers, we used an omics approach including iTRAQ, Oncomine (https://www.oncomine.org/resource/login.html), and the Plasma Proteome Database (http://www.plasmaproteomedatabase.org/) (Figure 1A). iTRAQ analyses revealed significant upregulation of oncogenic DEK in GC relative to paired normal tissues. The mean fold change in DEK expression in GC tissues was 1.94-fold higher than that in paired normal tissue in terms of relative expression (Table S1, Supplementary Materials). In addition, the evaluation of three public databases, specifically, the Oncomine public Chen, Cho, and DErrico Gastric datasets, consistently revealed significant overexpression of DEK in GC tissues relative to paired normal tissues (Figure S1, Supplementary Materials). Followed on the same selection criteria, DEK was worked further for verification in GC tissues and plasma specimens. In qRT-PCR analysis using 72 paired GC tissues, median DEK levels in normal and GC tissues (*n* = 72) were −17.25 and −16.22 (interquartile range, −10.479/−29.943 and −11.17/−24.971), respectively, significantly elevated in GC compared with normal gastric mucosa (*p* = 0.0059; Figure 1B). It is consistent with iTRAQ and public Oncomine data. The mean fold change in DEK expression in GC tissues was 14.87-fold (T > *n* = 47/72 = 65.28%, range: 0.004–204.8) than that in matched nontumorous gastric mucosa (Figure 1C).

### 2.2. Clinicopathologic Correlations of DEK in Gastric Tissues by IHC Study

**Figure 2:**
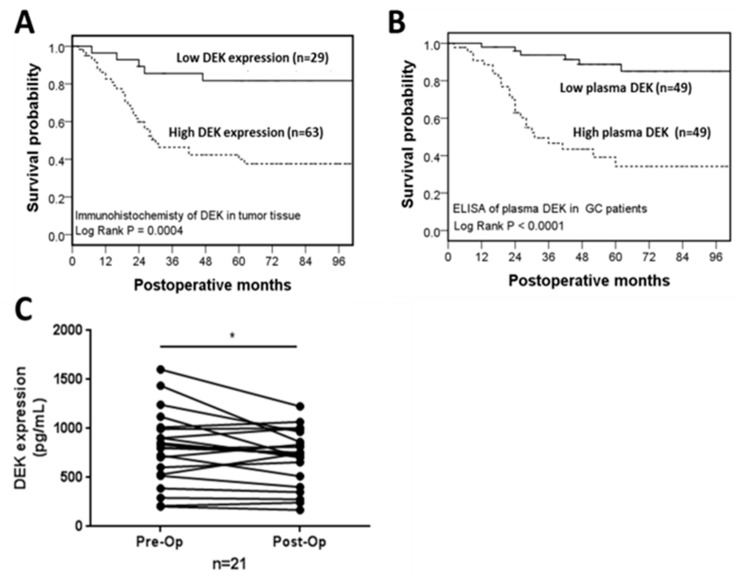
Kaplan–Meir survival curves of GC patients in two divided groups, high and low expressions, according to the IHC staining and plasma level in 98 GC patients. (**A**) DEK IHC staining in tumor tissues (positive stained cells: “<51%” vs. “≥51%”) (**B**) Plasma DEK level in GC patients (“<median” vs. “≥median”). (**C**) Plasma DEK level in 21 GC cases from the 98 original GC patients (paired pre- and postoperative samples), surgical removal of the tumor using paired sample *t*-tests.

DEK in gastric tissues was studied by IHC of the paraffin-fixed sections of gastrectomized specimens. Table 1 shows the correlation of tissue DEK with various clinicopathological characteristics in gastric tissues: gross type (*p* < 0.0001), size (*p* < 0.0001), depth of invasion (*p* < 0.0001), serosal invasion (*p* < 0.0001), lymph node status (*p* < 0.0001), lymph node metastasis (*p* < 0.0001), distant metastasis (*p* = 0.001), pathological stage (*p* < 0.0001), peritoneal seeding (*p* = 0.0312), lymphatic invasion (*p* < 0.0001), and perineural invasion (*p* = 0.0133). DEK expressions were compared between GC and adjacent normal tissues from stages I to IV (Figure 1D). Notably, DEK expression displayed a stepwise increase parallel to GC progression from the early to late stages. The distributions of IHC scores were as follows: “++” (29/92; 31.5%) and “+++” (63/92; 68.5%) in GC tissues, and “+” (2/90; 2.2%) and “++” (88/90; 97.8%) in adjacent nontumor tissues (Table 2). This finding additionally showed that DEK is strongly upregulated in GC tissues and stepwise increased from early to advanced stages. The DEK expressions were divided into two groups based on IHC scoring: IHC-low (<51% of cells with positive staining, or < “+++”) and IHC-high (≥51% of cells with positive staining, or ≥ “+++”). The five-year survival rate of the low DEK expression group was significantly better than that of the high DEK expression group (81.7% vs. 40.0%, log-rank *p* = 0.0004) (Figure 2A, Table 1), supporting a role of DEK as an oncoprotein during GC tumorigenesis. In view of these findings, we propose that DEK may serve as a novel prognostic factor influencing survival in GC patients.

### 2.3. Correlation of Plasma DEK with Clinicopathological Characteristic and Survival Outcome in GC Patients

A high expression of plasma DEK was significantly associated with the clinical and pathological characteristics of tumor progression, metastasis, or advanced stage of GC, manifested in the parameters: gross type, tumor size, histological type, depth of invasion, serosal invasion, lymph node metastasis, distant metastasis, pathological stage, liver metastasis, peritoneal seeding, vascular invasion, lymphatic invasion, and perineural invasion (Table 3). Figure 2B depicts the cumulative survival curves of the low and high plasma DEK expression groups, subdivided according to a median value of 734.0 pg/mL. The five-year survival rate of the low DEK expression group was significantly greater than that of the high expression group (*p* < 0.0001; Figure 2B; Table 3). Multivariate analysis was further performed to determine the independent potential of plasma DEK for GC prognosis in association with significant clinicopathological parameters identified in the univariate analysis. It revealed plasma DEK emerged as an independent prognostic biomarker for GC (*p* = 0.035, HR = 3.061, 95% CI = 1.079–8.682; Table 4). We speculate that a larger GC tumor burden may promote the release of a greater amount of plasma DEK from cancer cells into the bloodstream, when the disease is in progression. Thus, plasma DEK level is elevated in parallel to both tumor stage and progression of GC.

In 21 available patients from the 98 original GC patients, plasma DEK was measured both before and after gastrectomy. Plasma DEK was significantly reduced in 13 patients after gastric resection (*p* = 0.0427 by paired sample *t*-test) (Figure 2C).

### 2.4. Plasma DEK as a Potential Diagnostic Biomarker in GC Patients

**Figure 3:**
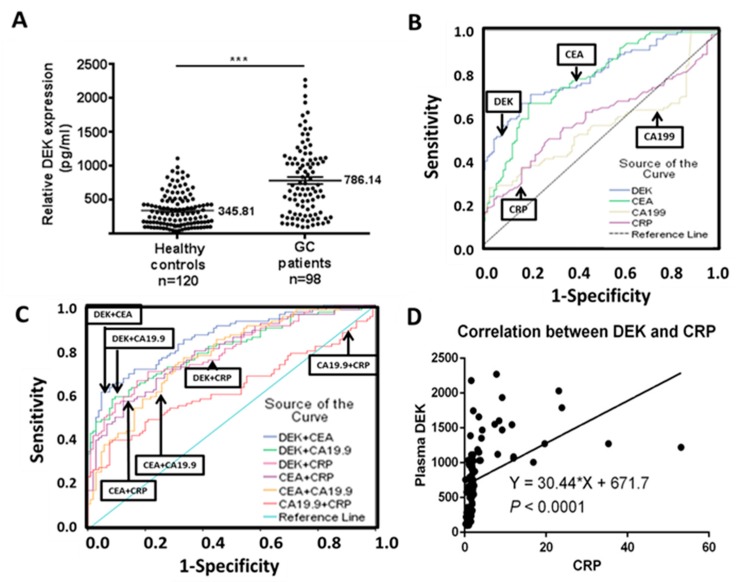
DEK levels in plasma from GC patients. (**A**) ELISA determination of plasma DEK levels in 120 healthy controls and 98 GC patients. (**B**) ROC curve analysis of DEK, CEA, and CA19.9 for discrimination between 98 GC patients and 120 healthy controls. AUC, area under the ROC curve. Logistic regression models were used for all pairwise comparisons. (**C**) ROC curve analysis of two combined in DEK, CEA, and CA19.9 for discrimination between 98 GC patients and 120 healthy controls. (**D**) Pearson’s correlation scatter plot of plasma DEK with plasma CRP in 98 GC patients (*p* < 0.0001).

As shown in Figure 3A, plasma DEK levels of GC patients (mean = 786.14 pg/mL) were significantly higher than for healthy controls (mean = 345.81 pg/mL) (*p* < 0.0001). Furthermore, for evaluating the diagnostic accuracy or discrimination between GC patients and healthy controls, ROC analysis of the four biomarkers were plotted. The areas under ROC curves (AUC) were calculated: 0.797 for DEK, 0.770 for CEA, 0.593 for CRP, and 0.555 for CA19.9 (Table 5; Figure 3B). AUC can provide a rough guide for classifying the accuracy of a diagnostic test in the traditional academic point system: 0.90–1.0 = excellent; 0.80–0.90 = good; 0.70–0.80 = fair; 0.60–0.70 = poor; 0.50–0.60 = fail [27]. Accordingly, both DEK and CEA would be classified as “fair”, whereas both CRP and CA19.9 would be “fail”. Pairwise comparison of AUCs between biomarkers revealed that both DEK and CEA are comparable in AUC (*p* = 0.5493), and both are superior to CA19.9 (*p* < 0.0001) and CRP (*p* < 0.0001) in terms of diagnostic accuracy (Table 6). If plasma DEK was combined with CEA or CA19.9, the diagnostic accuracy could be upgraded (AUC = 0.855 and 0.802, respectively), from “fair” into the “good” category (AUC > 0.8); however, the improvement was not statistically significant from a single use of plasma DEK (*p* = 0.1483, *p* = 0.9089, and *p* = 0. 9819, respectively; Figure 3C, Tables S2 and S3).

The sensitivity and specificity that derived from the ROC analysis are also shown in Table 5. Plasma DEK has better sensitivity for GC diagnosis than the other three biomarkers (*p* < 0.0001 for CA19.9 and *p* = 0.0205 for CRP), but it was not significant for CEA (*p* = 0.5388) (Table 4 and Table 5). The specificity of plasma DEK was inferior to CA19.9 (*p* = 0.0003), but it was not significantly different from CEA and CRP (Table 5 and Table 6). However, if the cut-off values of the four biomarkers in ROC analysis were changed by using the upper limit (97.5 percentile) of reference ranges of the healthy controls to calculate the sensitivity, the upper limit of the reference range in our 120 healthy controls would be the following: 846.63 pg/mL for DEK, 3.65 ng/mL for CEA, 23.42 U/mL for CA19.9, and 3.62 mg/L for CRP, respectively, and the sensitivity of the plasma biomarkers would be changed accordingly as in Table 7. For GC diagnosis, the sensitivity of DEK was significantly superior to the other three biomarkers, CEA, CRP, and CA19.9 (*p* = 0.0063, *p* = 0.0363 and *p* = 0.0004, respectively, whereas the specificity of DEK did not differ significantly from the other three biomarkers (Table 8).

Notably, a moderately positive correlation was evident between plasma DEK and CRP levels (*r* = 0.445, *p* < 0.0001; Figure 3D). Moreover, among the four plasma markers, both DEK and CRP had a prognostic effect on patients’ survival in the univariate analysis (Table 3). CRP also had a higher sensitivity than CA19.9 in the ROC analysis (*p* = 0.0013).

### 2.5. DEK Is Involved in the Invasive Ability of GC Cell Lines

**Figure 4:**
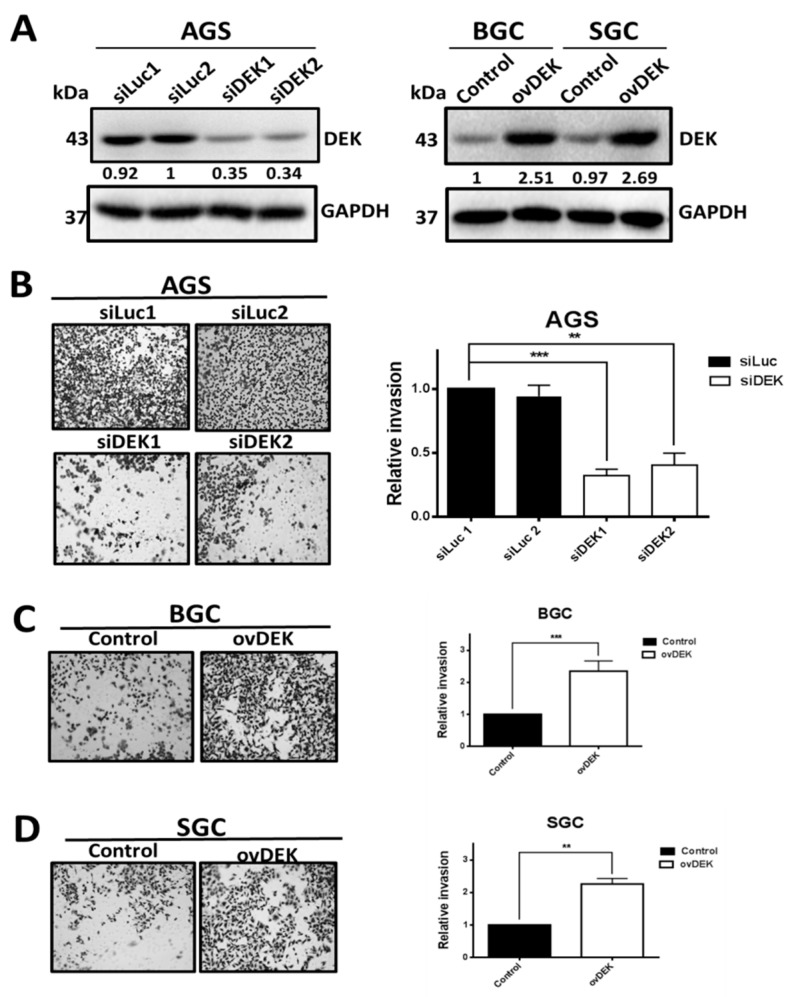
DEK contributes to the invasive ability of GC cells. (**A**) Knockdown of DEK inhibits the invasive ability of the AGS cell line. AGS cells were transfected with control and DEK siRNA, respectively. After two days, cell lysates were prepared and the extracted proteins (50 μg) analyzed via Western blot (**left**). Moreover, overexpression of DEK enhances the invasive abilities of BGC and SGC cells. The two GC cell lines were transfected with control pcDNA and pcDEK. After two days, cell lysates were prepared, and the extracted proteins (50 μg) analyzed via Western blot (**right**). (**B**) Invasion assay of GC cell lines. Representative microphotographs of filters obtained from the invasion assay are shown, along with quantitative analysis. (**C**,**D**) Invasion assay of cell lines as described in Materials and Methods. Representative microphotographs of filters obtained from the invasion assay are shown, along with quantitative analysis. Data are presented as mean values of cell counts obtained from three independent experiments. A *p*-value less than 0.05 indicates statistical significance according to the Mann–Whitney *U* test (* *p* < 0.01, ** *p* < 0.05, *** *p* < 0.001).

To explore the potential role of DEK in GC cell invasion, cells were transfected with shDEK plasmid to eliminate endogenous DEK expression in AGS cell lines and the consequent effects on invasive activity assessed. In Western blot experiments, DEK protein levels were clearly reduced in AGS cells transfected with shDEK1 and shDEK2, compared with those transfected with shLuc control, as shown in Figure 4A, left. Notably, the invasion ability of DEK-knockdown AGS cell lines was reduced to 0.34- and 0.37-fold that of control GC cells, respectively (Figure 4B). To further establish whether overexpression of DEK conversely increases invasive ability, BGC and SGC cell lines were transfected with pcDNA3-DEK plasmid (Figure 4A, right). As shown in Figure 4C,D, overexpression of DEK promoted the invasive ability of BGC and SGC by 2.31- and 2.26-fold, respectively, compared to cells transfected with the pcDNA3 control vector.

### 2.6. DEK Promotes GC Cell Metastasis Mediated by MMP-2 and MMP-9

**Figure 5:**
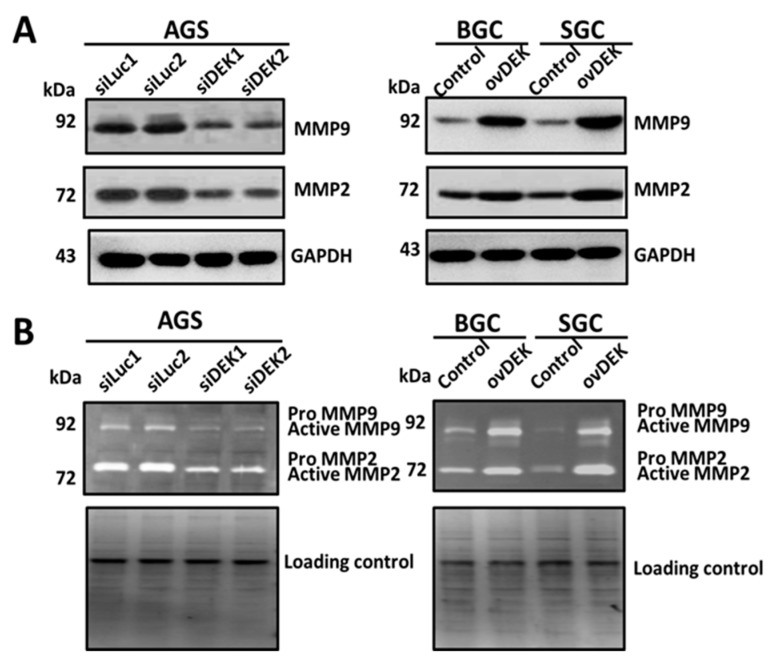
DEK induces MMP-9/MMP-2 level. (**A**) Total cell lysates from AGS, BGC, and SGC cells were analyzed via Western blot to detect MMPs. Actin served as an internal control. (**B**) Zymography revealing MMP-2 and MMP-9 expression in AGS, BGC, and SGC cells. Conditioned media from the three cell lines were assayed for MMP level as described in Materials and Methods. The electrophoretic positions of both proenzymes and active MMPs are shown.

Overexpression of the oncogene DEK may induce an autocrine or paracrine mechanism through the release of several proinflammatory factors (IL-8, c-IAP2, MCP-1/CCL-2) during tumorigenesis and chronic inflammation [28]. Based on previous studies, we examined the interactions between DEK protein and other factors involved in the inflammatory pathway, including MMP-2, MMP-9, p53, VEGF, HIF-1α, and RhoA. Notably, p53, VEGF, HIF-1α, and RhoA levels in cell lines stably expressing DEK remained unchanged, as determined via Western blot (Figure S2), whereas MMP-2 and MMP-9 expression was significantly increased in AGS but decreased in BGC and SGC cell lines (Figure 5A). Gelatin zymography further showed that MMP-2 and MMP-9 level increased upon expression of DEK in AGS cells but decreased in BGC and SGC cells overexpressing DEK (Figure 5B). The collective results indicate that DEK participates in cell invasion during tumorigenesis of GC via a mechanism involving MMP-2 and MMP-9.

## 3. Discussion

Detection of conventional serum tumor biomarkers (CEA, CA19.9, and CA72.4) is commonly applied for diagnosis and follow-up of GC [29]. The identification of novel biomarkers for GC is a feasible option to improve treatment outcomes [30]. Both genomic and proteomic approaches have recently been applied to understand global proteomic dynamics in tissue and plasma specimens. Importantly, research on the human plasma proteome database indicates that DEK is released into the blood, a common phenomenon in GC tissues, which further supports the theory that proteomics presents an efficient method to discover potentially useful biomarkers for GC.

In this study, plasma DEK (AUC = 0.797) displayed higher diagnostic accuracy than CEA (AUC = 0.770), CRP (AUC = 0.593), and CA19.9 (AUC = 0.555). Accordingly, the diagnostic accuracy of DEK was classified as “fair”, a little below that required for a “good” grade (AUC > 0.8) in ROC analysis highlighting the need for additional studies with larger sample sizes. Notably, plasma DEK combined with CEA could escalate the diagnostic accuracy into a good grade (AUC > 0.8) (Figure 3C and Table S2). CEA and CA19.9 are the two most commonly used biomarkers of GC with reported sensitivity and specificity of ≈30% and 72%–95%, respectively [4,31]. The sensitivity and specificity of plasma DEK in our series were 42.9% and 64.1%, respectively, based on the cut-off value (846.63 pg/mL) at the upper limit of reference range in our 120 healthy controls (Table 7). Plasma DEK would show a higher sensitivity than CEA, CA19.9, and CRP in the diagnosis of GC. Moreover, both CRP and DEK could be induced and secreted into the blood stream in response to inflammation [5,28,32]. Our data indicate a positive association between plasma DEK and CRP in GC patients. The superiority of plasma DEK over CEA, CA19.9, and CRP in diagnostic accuracy supports the clinical usefulness of plasma DEK in the detection of malignancy. DEK has been investigated as a potential tissue biomarker for many human cancers to date [16,28,33].

In this study, we selected DEK for the biological function study in GC, due to its upregulation in both tumor and blood stream. Furthermore, involvement of DEK in several signaling pathways, including p53, NF-κB, Wnt, mTOR, and Rho, has been reported [28,32,34,35], although the precise signaling mechanism underlying its expression remains to be established. DEK is actively secreted in both free form (non-classical) and exosome (classical) [8], which could be detectable in urine (bladder cancer) [11], plasma (oropharyngeal) [36], synovial fluid (juvenile idiopathic arthritis (JIA)) [26,37], and the HepG2 cell line (conditioned medium) [22,23]. Macrophages are not the only cells that secrete DEK in association with poor prognosis [26]. In vitro, DEK is polyADP-ribosylated and released into the extracellular space by apoptotic cells [38]. These results highlight a potential role of extracellular DEK in stimulating tumor-associated immunological responses and intracellular oncogenic activity in adjacent epithelial cells within the tumor microenvironment. To further confirm that DEK in plasma is of tumoral origin, a complex response process that transfers changes from tissue to the circulating system is essential. Accordingly, we compared plasma DEK before and after gastrectomy in a postoperative follow-up survey in 21 GC patients from the 98 original GC patients (Figure 2C). Plasma DEK level was significantly reduced in 13 patients (*p* = 0.0427, by paired sample *t*-test). These data suggest that plasma DEK may be tumor-derived and have the potential to be a novel biomarker for monitoring GC dynamics. However, immune cells in the tumor microenvironment may also affect plasma DEK concentrations through the bloodstream. DEK may mediate inflammation and immunity responses in tumor microenvironments. On the other hand, our data showed a positive correlation between intratumoral DEK staining scores and plasma DEK concentrations, suggesting that a large tumor burden promotes release of DEK from cancer cells. The implications of DEK activity in the context of tumorigenic microenvironments are poorly understood at present. Data from the current study provide potential insights that should aid in elucidating the mechanisms underlying regulation of GC tumorigenesis by DEK.

You et al. [39] demonstrated that the epithelial–mesenchymal transition (EMT) of colorectal carcinoma cells is partially mediated by DEK-regulated E-cadherin, vimentin, and matrix metalloproteinase (MMP-9). Another group showed that silencing of DEK led to downregulation of Wnt/β-catenin and MMP-9 in cervical cancer [13]. Experiments on a rat model by Sadeeshkumar et al. [40] additionally disclosed that DEK modulates the expression of key molecules that regulate apoptosis, inflammation, invasion, and angiogenesis (MMP-2/-9, VEGF). Furthermore, another group reported that microRNA-1292–5p directly targets DEK-mediated migration, invasion, and cell growth [41].

In summary, plasma DEK is superior to CEA, CA19.9, and CRP in terms of the sensitivity for the detection of GC. Its diagnostic accuracy (=0.797) by ROC analysis is still not satisfactory for clinical practice, unless it can be classified into the “good” level (=0.800). Nevertheless, it may serve as a novel biomarker effective for monitoring the progression or metastasis in GC patients. Further research, preferably with the aid of large prospective studies, is warranted, before DEK can be clinically applied as a non-invasive screening marker and therapeutic target for GC.

## 4. Materials and Methods

This study intended to identify useful biomarkers in both tumor tissues and peripheral bloods from 98 GC patients and 120 healthy controls.

### 4.1. Clinical Specimens

The study was approved by the Institutional Review Board (IRB No. 103–7252B) of Chang Gung Memorial Hospital (CGMH). The specimens consisted of (1) fresh plasma from presurgical GC patients and healthy controls and (2) fresh stomach tissues surgically removed (gastrectomy) from GC patients. Both were frozen and stored in the Biobank of Chang Gung Memorial Hospital.

Whole blood of GC patients was withdrawn one day before surgery. Blood was centrifuged at 12,000 rpm for 15 min and isolated to collect plasma samples, which were frozen at −70 °C until use. All patients were pathologically diagnosed with gastric carcinoma and underwent gastric resection at CGMH of Chiayi. Tissue specimens were obtained from 92 patients (51 males and 41 females; median age of 67.5 years, range 28–84 years) who underwent surgery between 2001 and 2008.

Plasma specimens were obtained before surgery from 98 patients (58 males and 42 females; median age of 66.6 years, range 28–87 years) who also underwent gastrectomy between 2004 and 2012. The demographic characteristics of patients who contributed both tissue and plasma specimens are presented in Table 1 and Table 3, respectively. Frozen plasma was additionally donated by 120 healthy control volunteers from the CGMH Healthcare Center (54 males and 66 females) between 2016 and 2017 for analysis. Further, plasma DEK was measured both before and after gastrectomy in 21 available patients from the 98 original GC patients (Figure 2C).

### 4.2. Statistical Analysis

The Mann–Whitney *U* test (for two groups), the Kruskal–Wallis test (for more than two groups), or Fisher’s exact test was performed for between-group comparisons. The correlation between two paired variables was investigated using bivariate Pearson’s correlation coefficient. For determining prognostic significance, the cumulative 5-year survival rates of all GC patients were calculated using the log-rank test (excluding patients who died from diseases other than GC) to compare survival distribution of the groups. Cox’s proportional hazards model was applied as a multivariate analysis to identify independent predictors of survival. For diagnostic test evaluation, we applied SPSS software to plot receiver operating characteristic (ROC) curves that would generate an area under the curve (AUC) and its statistical significance. For combined ROC analysis, we applied the binary logistic regression to calculate the covariates for combined biomarkers according to their plasma levels, and then used the results as the probability to plot ROC curves [42]. Statistical analyses were performed using SPSS software (Version 19.0, SPSS Inc., Chicago, IL, USA). Data were considered statistically significant at *p*-values <0.05.

### 4.3. Cell Culture

Human GC cell lines (AGS) obtained from the American Type Culture Collection (ATCC, CRL-1739, Manassas, WV, USA) as well as BGC and SGC (Xiamen, China) were incubated in Roswell Park Memorial Institute (RPMI) medium 1640 (Invitrogen, Waltham, MA, USA) with 10% fetal bovine serum (FBS) plus 100 IU/mL penicillin G and 100 mg/mL streptomycin sulfate (Sigma-Aldrich, St Louis, MO, USA) and nonessential amino acids (NEAA) at 37 °C in 95% air and 5% CO_2_ [43].

### 4.4. Measurement of CEA, CA19.9, and CRP Levels

Plasma CEA, CA19.9, and CRP levels were detected via fully automated electrochemistry luminescence immunity analyzer with the Roche Modular E170 platform (Roche Diagnostics, Mannheim, Germany) in the Department of Laboratory Medicine at CGMH [43].

### 4.5. qRT-PCR

Total RNA was extracted via TRIzol reagent (Invitrogen, Carlsbad, CA, USA) from surgical specimens. The concentrations of all RNA samples were determined using a Colibri Microvolume Spectrometer (Pforzheim, Germany) [44]. To evaluate DEK mRNA expression in GC tissues, qRT-PCR was performed as described previously, using 18sRNA as an internal control for DEK [44,45]. Fluorescence emitted by SYBR Green was assayed using the ABI PRISM 7500 sequence detection system (Applied Biosystems, Werrington, UK). The primers used for RT-qPCR were as follows: DEK (forward, 5′–CAAAGCCTTCTGGCAAACCA–3′, and reverse, 5′–CCTTGCCATTCCAGAACTGTTC–3′) and human 18s rRNA (forward, 5′–CGAGCCGCCTGGATACC–3′, and reverse, 5′–CCTCAGTT CCGAAAACCAACAA–3′).

### 4.6. Western Blot

Total cell extracts were subjected to 12% SDS-PAGE and separated proteins transferred to polyvinylidene fluoride membrane. Western blot was performed using standard protocols described previously [46]. Rabbit anti-DEK, anti-E-cadherin, anti-vimentin, anti-MMP2, and anti-MMP9 antibodies were purchased from Proteintech (Chicago, IL, USA). Mouse anti-GAPDH antibody was obtained from Chemicon (Temecula, CA, USA).

### 4.7. ELISA

Plasma DEK protein levels were measured using the Human Protein DEK ELISA Kit (MyBiosource, San Diego, CA, USA). All plasma samples were diluted 1:8 in sample dilution buffer, and various amounts of DEK recombinant protein added to the wells. Fluorescence intensity was measured at 450 nm using a SpectraMax M5 microplate reader (Molecular Devices, Sunnyvale, CA, USA).

### 4.8. Overexpression/Depletion of DEK in GC Cell Lines

For overexpression purposes, DEK cDNA was amplified using RT-PCR and cloned into pcDNA3 [43]. Transfection of pcDNA3-ovDEK or pcDNA3-control vector into GC cell lines was performed using TurboFect Reagent (Invitrogen, Grand Island, NY, USA). After 24 h of incubation, cells were transferred to G418 medium for selection and cell lysates subjected to Western blot after two weeks to determine gene overexpression efficacy. Alternatively, DEK knockdown was performed with the aid of specific shRNA. Clones of DEK-targeting shRNA (shDEK1, TRCN0000235737; shDEK2, TRCN0000235740) and shRNA-Luc control were purchased from the National RNA Interference Core Facility (Academia Sinica, Taiwan). Transfection of shRNAs targeting endogenous DEK genes into GC cell lines was performed using TurboFect Reagent (Invitrogen, Grand Island, NY, USA). After 24 h of incubation, cells were selected in the presence of puromycin for two weeks of selection, followed by Western blot analysis of lysates to determine gene knockdown efficacy.

### 4.9. In Vitro Invasion Assay

To assess the influence of DEK overexpression or depletion in GC cell lines on metastatic activity, the in vitro Transwell assay (Becton-Dickinson, Franklin Lakes, NJ, USA) was employed. After adjusting the density to 1 × 10^5^ cells/100 μL serum-free RPMI, cells were added to the upper chamber and a Matrigel-coated (invasion assay) insert used to assess invasive capability, as described previously [46].

### 4.10. Immunohistochemistry (IHC) and Scoring

Paraffin-embedded tissues (5 μm thick) were prepared for different GC tissue specimens and IHC performed to detect DEK (Epitomics, Burlingame, CA, USA; dilution 1:150), as described previously [47]. The intensity and percentage of staining of the entire tissue section per specimen (200× magnification) was evaluated. Samples with ≥51% positively or strongly stained tumor cells were denoted “High” and those showing positive staining for <51% tumor cells denoted “Low” in IHC analyses. Alternatively, IHC scores were assigned 0 (<1% positive cells), 1+ (1%–10% positive cells), 2+ (11%–50% positive cells) or 3+ (≥51% positive cells).

### 4.11. Gelatin Zymography

Conditioned medium from various GC cell lines was harvested and concentrated in the absence of reducing agent, in keeping with a previously reported protocol [47].

## Figures and Tables

**Table 1:** Clinicopathological correlations of DEK expression in cancer tissue and 5-year survival rate (S.R.) in 92 GC patients.

Characteristics	No.	High Expression ^a^ No. (%)	*p*-Value ^b^	5-yr S.R. ^c^	Log-Rank *p* ^d^
Age (yrs)					
<65	40	27 (67.5%)	1.000	51.6	0.5487
≥65	52	36 (69.2%)		55.4	
Gender					
Male	51	36 (70.6%)	0.6574	54.0	0.9059
Female	41	27 (65.9%)		53.4	
Location					
Upper third	21	14 (66.7%)	0.5558	57.4	0.9837 ^f^
Middle third	21	12 (57.1%)		58.7	
Lower third	45	33 (73.3%)		56.1	
Whole	5	4 (80.0%)		0.0	
Gross type					
Localized	36	15 (41.7%)	<0.0001	82.1	<0.0001
Infiltrative	56	48 (85.7%)		34.5	
Size (maximal diameter)					
<5 cm	51	26 (51.0%)	<0.0001	77.2	<0.0001
≥5 cm	41	37 (90.2%)		20.3	
Histological type					
Intestinal	26	14 (53.8%)	0.0809	82.9	0.0011
Diffuse	66	49 (72.4%)		40.2	
Depth of invasion (pT)					
T1	19	2 (10.5%)	<0.0001	94.1	<0.0001
T2	13	9 (69.2%)		72.5	
T3	41	35 (85.4%)		42.7	
T4	19	17 (89.5%)		7.9	
Serosal invasion					
No (T1, T2)	32	11 (34.4%)	<0.0001	85.9	<0.0001
Yes (T3, T4)	60	52 (86.7%)		31.9	
Lymph node status (pN)					
N0	30	9 (30.0%)	<0.0001	93.1	<0.0001
N1	34	28 (82.4%)		48.0	
N2	18	16 (88.9%)		17.1	
N3	10	10 (100.0%)		0.0	
Lymph node metastasis					
No	30	9 (30.0%)	<0.0001	93.1	<0.0001
Yes	62	54 (87.1%)		32.1	
Distant metastasis (pM)					
No	75	46 (61.3%)	0.0010	67.2	<0.0001
Yes	17	17 (100.0%)		0.0	
Pathological stage (pStage)					
Stage I	23	4 (17.4%)	<0.0001	100.0	<0.0001
Stage II	12	7 (58.3%)		68.8	
Stage III	37	33 (89.2%)		42.3	
Stage IV	20	9 (95.0%)		0.0	
Pathological stage					
Stage I, II	35	11 (31.4%)	<0.0001	90.1	<0.0001
Stage III, IV	57	52 (91.2%)		25.2	
Liver metastasis					
No	89	60 (67.4%)	0.5490	55.3	0.0030
Yes	3	3 (100.0%)		0.0	
Peritoneal seeding					
No	77	49 (63.6%)	0.0312	65.9	<0.0001
Yes	15	14 (93.3%)		0.0	
Vascular invasion					
No	68	45 (66.2%)	0.6099	64.7	0.0001
Yes	24	18 (75.0%)		19.9	
Lymphatic invasion					
No	38	17 (44.7%)	<0.0001	79.6	<0.0001
Yes	54	46 (85.2%)		35.7	
Perineural invasion					
No	52	30 (57.7%)	0.0133	68.8	0.0008
Yes	40	33 (82.5%)		32.4	
DEK ^(e)^ (IHC) expression					
Low	29			81.7	0.0004
High	63			40.0	

**^a^** High expression of DEK IHC staining: positive rate ≥51% of tumor cells. **^b^** Fisher’s exact test (for two groups) or chi-squared test (for more than two groups). **^c^** Five-year survival rate. **^d^** Log-rank test. **^e^** “Low”: positive staining rate <51% of tumor cells; “High”: positive staining rate ≥51% of tumor cells. **^f^** If “whole” is not included.

**Table 2:** IHC scores for DEK expression in tumor tissues and adjacent nontumor tissues of the GC patients.

Tissues	No. Patients	DEK Staining Score ^a^/No. Patients (%)			
		-	+	++	+++
Tumor	92	0	0	29 (31.5%)	63 (68.5%)
Adjacent mucosa	90	0	2 (2.2%)	88 (97.8%)	0

**^a^** IHC staining score: “-” (<1% positive cells); “+” (1%–10% positive cells); “++” (11%–50% positive cells); “+++” (≥51% positive cells).

**Table 3:** Correlation of clinicopathological characteristics and 5-year survival rates with plasma DEK expressions in 98 GC patients.

Clinicopathological Correlations				Univariate Analysis	
Characteristics	No.	Mean ± SE Plasma DEK (pg/mL) ^a^	*p* ^b^	5-yr S.R. ^c^	Log-Rank *p* ^d^
Age (yrs)					
<65	45	762.4 ± 79.1	0.4205	60.1	0.2335
≥65	53	820.7 ± 69.3		67.2	
Gender					
Male	56	751.3 ± 61.5	0.5346	69.8	0.1061
Female	42	850.8 ± 88.6		56.4	
Location					
Upper third	21	816.2 ± 105.2	0.7472	54.6	0.3590 ^(f)^
Middle third	23	795.1 ± 120.5		75.6	
Lower third	50	770.0 ± 71.6		68.7	
Whole	4	987.5 ± 283.4		0.0	
Gross type					
Localized	47	584.3 ± 78.1	<0.0001	86.4	<0.0001
Infiltrative	51	987.1 ± 58.0		42.7	
Size (maximal diameter)					
<5 cm	55	613.6 ± 57.3	<0.0001	84.8	<0.0001
≥5 cm	43	1024.5 ± 81.2		34.3	
Histological type					
Intestinal	28	554.9 ± 72.4	0.0058	92.6	0.0016
Diffuse	70	889.5 ± 63.5		53.2	
Depth of invasion (pT)					
T1	24	311.4 ± 58.6	<0.0001	93.3	<0.0001
T2	17	774.2 ± 151.8		86.3	
T3	42	909.8 ± 61.1		58.0	
T4	15	1263.6 ± 136.6		0.0	
Serosal invasion					
No (T1, T2)	41	503.3 ± 76.9	<0.0001	90.5	<0.0001
Yes (T3, T4)	57	102.9 ± 56.2		42.1	
Lymph node status (pN)					
N0	37	505.8 ± 80.2	<0.0001	91.9	<0.0001
N1	36	893.9 ± 70.0		65.9	
N2	17	1065.1 ± 126.0		21.6	
N3	8	1100.1 ± 148.8		14.3	
Lymph node metastasis					
No	37	505.8 ± 80.2	<0.0001	91.9	0.0001
Yes	61	868.7 ± 57.7		46.3	
Distant metastasis (pM)					
No	81	674.7 ± 49.8	<0.0001	77.0	<0.0001
Yes	17	1362.1 ± 105.7		0.0	
Pathological stage (pStage)					
Stage I	30	375.2 ± 79.3	<0.0001	100.0	<0.0001
Stage II	12	752.4 ± 150.4		75.8	
Stage III	37	872.7 ± 44.5		54.0	
Stage IV	19	1327.8 ± 104.1		0.0	
Pathological stage					
Stage I, II	42	483.0 ± 75.0	<0.0001	92.6	<0.0001
Stage III, IV	56	1027.1 ± 53.9		35.6	
Liver metastasis					
No	97	968.1 ± 52.2	0.2095	64.9	0.0022
Yes	1	1354.1		0.0	
Peritoneal seeding					
No	83	717.4 ± 53.3	0.0006	74.0	<0.0001
Yes	15	1217.3 ± 123.9		14.3	
Vascular invasion					
No	71	678.8 ± 57.3	0.0003	75.0	0.0001
Yes	27	1096.5 ± 92.5		34.4	
Lymphatic invasion					
No	43	576.0 ± 71.3	0.0001	88.1	0.0001
Yes	55	964.2 ± 65.9		44.7	
Perineural invasion					
No	54	600.3 ± 64.4	<0.0001	80.7	0.0001
Yes	44	1031.5 ± 72.3		42.1	
DEK (Plasma)					
<median ^(e)^ (=734.0 pg/mL)	49			88.7	<0.0001
≥median	49			34.2	
CEA (Plasma)					
<median (=2.11 ng/mL)	49			57.1	0.1442
≥median	49			67.6	
CA19.9 (Plasma)					
<median (=9.91 U/mL)	49			70.2	0.3420
≥median	49			57.9	
CRP (Plasma)					
<median (=1.79 mg/L)	49			83.9	<0.0001
≥median	49			41.7	

**^a^** Plasma DEK (pg/mL) as the mean ± SE by ELISA. **^b^** Mann–Whitney *U* test (for two groups) or Kruskal–Wallis test (for more than two groups). **^c^** Five-year survival rate. **^d^** Log-rank test. **^e^ =** 50th percentile. **^f^** If “whole” not included.

**Table 4:** Multivariate survival analyses of various characteristics of 98 GC patients using a Cox regression model.

Characteristics	B ^a^	SE ^b^	Wald	HR ^c^	95% CI ^d^	P ^e^
Histological type (intestinal/diffuse)	1.341	1.113	1.451	3.824	0.431–33.902	0.228
Gross type (localized/infiltrative)	0.442	0.661	0.446	1.555	0.425–5.687	0.504
Tumor size (<5 cm/≥5 cm)	0.644	0.653	0.974	1.905	0.530–6.851	0.324
Serosal invasion (no/yes)	−0.085	0.820	0.011	0.918	0.184–4.581	0.917
Lymph node metastasis (no/yes)	0.176	1.072	0.027	1.192	0.146–9.742	0.870
Distant metastasis (no/yes)	0.102	0.623	0.027	1.107	0.326–3.754	0.871
Liver metastasis (no/yes)	3.363	1.848	3.312	28.887	0.772–1081.15	0.069
Pathological stage (I, II/III, IV)	1.375	1.096	1.573	3.955	0.461–33.926	0.210
Peritoneal invasion (no/yes)	0.301	0.668	0.203	1.351	0.365–5.007	0.652
Vascular invasion (no/yes)	0.268	0.464	0.333	1.307	0.526–3.247	0.564
Lymphatic invasion (no/yes)	−0.040	0.717	0.003	0.961	0.236–3.920	0.956
Perineural invasion (no/yes)	0.265	0.506	0.274	1.303	0.484–3.511	0.601
CRP (<median/≥median)	0.311	0.500	0.387	1.365	0.512–3.639	0.534
Plasma DEK level (<median/≥median)	1.119	0.532	4.421	3.061	1.079–8.682	0.035

**^a^** B coefficient **^b^** Standard error **^c^** Hazard ratio **^d^** Confidence interval **^e^** Cox regression model.

**Table 5:** The AUC, sensitivity and specificity of plasma DEK CEA, CA19.9, and CRP for diagnosis of GC, calculated from the ROC analysis.

Biomarkers	AUC ^a^	SE ^b^	*p*-Value ^c^	Cut-Off Value	Sensitivity ^d^	Specificity ^e^
DEK	0.797	0.031	<0.001	484.22 pg/mL	70.4%	79.0%
CEA	0.770	0.033	<0.001	1.90 ng/mL	66.3%	80.0%
CA19.9	0.555	0.042	0.1726	23.57 U/mL	27.6%	95.8%
CRP	0.593	0.041	0.0228	1.81 mg/L	50.0%	74.2%

**^a^** AUC, area under the ROC curve. **^b^** SE, standard error. **^c^** Fisher’s exact test. A *p*-value > 0.05 was not significant. **^d^** Sensitivity indicates proportion of plasma-positive patients among cancer patients. **^e^** Specificity indicates proportion of cancer-free participants among plasma-negative participants.

**Table 6:** Pairwise comparisons (in *p*-values) of AUC, sensitivity, and specificity of biomarkers for GC, based on the ROC curves.

Biomarkers	AUC ^a^	Sensitivity ^b^	Specificity ^b^
DEK vs. CEA	0.5493	0.5388	0.8573
DEK vs. CA19.9	<0.0001	<0.0001	0.0003
DEK vs. CRP	<0.0001	0.0035	0.4819
CEA vs. CA19.9	<0.0001	<0.0001	0.0004
CEA vs. CRP	0.0005	0.0205	0.3568
CA19.9 vs. CRP	0.4911	0.0013	<0.0001

**^a^***p*-values by online calculator: “comparison of Two ROC Curves—VassarStats”. **^b^***p*-values by chi-squared test.

**Table 7:** Sensitivity and specificity for the diagnosis of GC among 98 GC patients and 120 healthy controls. The 97.5 percentile was set as the upper limit of the reference range of the healthy controls in calculating the sensitivity and specificity of each plasma biomarker.

Plasma Biomarkers	Upper Limit of Reference Range	Sensitivity	Specificity
DEK	846.63 pg/μL	42.9%	64.1%
CEA	3.65 ng/mL	23.5%	64.3%
CA19.9	23.42 U/mL	27.6%	61.6%
CRP	3.62 mg/L	18.4%	59.0%

**Table 8:** Pairwise comparisons (*p*-value **^a^**) of sensitivity of plasma biomarkers between plasma biomarkers in 98 GC patients, based on the cut-off point set at the upper limit (97.5 percentile) of reference ranges of the healthy controls.

Biomarkers	Sensitivity
DEK vs. CEA	0.0063
DEK vs. CA19.9	0.0363
DEK vs. CRP	0.0004
CEA vs. CRP	0.6250
CEA vs. CA19.9	0.4824
CRP vs. CA19.9	0.1742

**^a^***p*-value by chi-squared test. Significant if <0.05.

## Supplementary Materials

Supplementary Figure and Tables can be found at https://www.mdpi.com/1422-0067/20/22/5689/s1.

## Abbreviations

AUC	Area under the curve
CA 19.9	Carbohydrate antigen 19.9
CEA	Carcinoembryonic antigen
CGMH	Chang Gung Memorial Hospital
CI	Confidence interval
CRP	C-reactive protein
ELISA	Enzyme-linked immunosorbent assay
GC	Gastric cancer
*Hp*	*Helicobacter pylori*
HR	Hazard ratio
IHC	Immunohistochemistry
iTRAQ	Isobaric tags for relative and absolute quantitation
qRT-PCR	Quantitative real-time polymerase chain reaction
MMP	Matrix metalloproteinase
ROC	Receiver operating characteristic
TNM stage	Tumor–node–metastasis stage
