# Supplementary material for: DEK Is a Potential Biomarker Associated with Malignant Phenotype in Gastric Cancer Tissues and Plasma
Source: Int J Mol Sci. 2019 Nov 13;20(22):5689. doi: 10.3390/ijms20225689 (PMC6888682; doi:10.3390/ijms20225689)
Supplement: Supplementary file 1 [file ijms-20-05689-s001.pdf]

Supplemental Figure and Tables

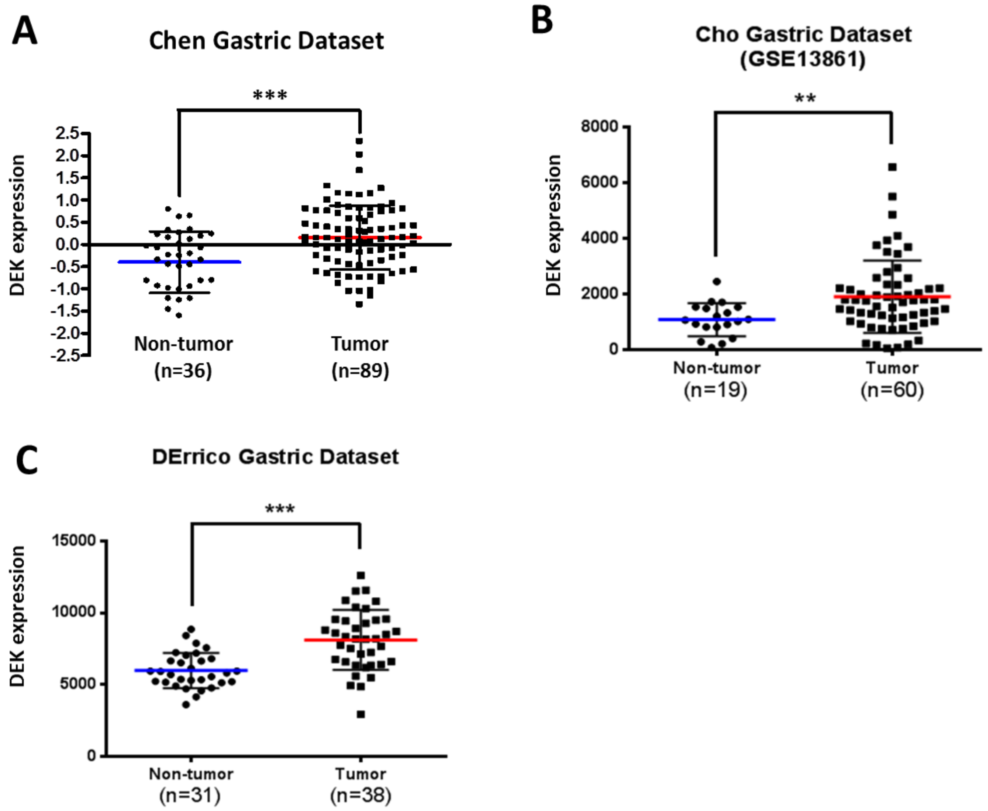

Figure S1. DEK expression is significantly increased in GC tissue according to the Oncomine database. Scatter plots of DEK expression in GC and adjacent normal tissues (n=72) determined via qRT-PCR. Error bars indicate standard deviations. **(A)** Chen Gastric Dataset, **(B)** Cho Gastric Dataset, **(C)** DErrico Gastric Dataset. Statistical analysis was performed using the Mann-Whitney U test (*, *p*<0.01, **, *p*<0.05, ***, *p*<0.001).

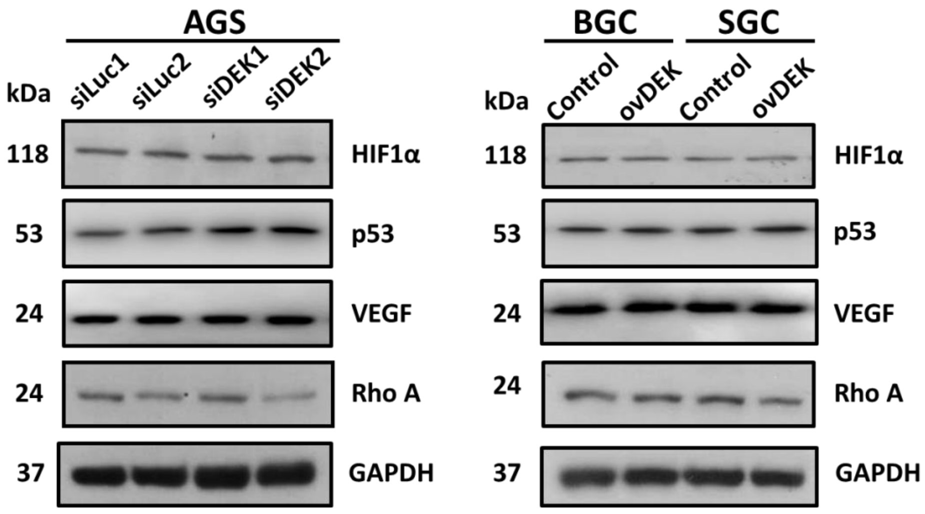

Figure S2. p53, VEGF, HIF-1α and RhoA expression levels in stably expressing knockdown or overexpression DEK GC cell lines. **(A)** Total cell lysates from knockdown of DEK of the AGS cell line. Cell lysates were prepared and the extracted proteins (50 μg) analyzed by western blot. **(B)** Overexpression of DEK in BGC and SGC cells. Cell lysates were prepared, and the extracted proteins (50 μg) analyzed by western blot. GAPDH served as an internal control.

Table S1. Comparison of the adjacent nontumor mucosa and the tumor tissue; the iTRAQ study of DEK expression in GC patients.

	**GC1**	**GC2**	**GC3**	**GC4**	**GC5**	**GC6**	**Mean**
**Lauren's type**	**Int.**[a]	**Int.**	**Int.**	**Dif.** [b]	**Dif.**	**Dif.**	
**DEK T/N Ratio**	1.88	2.02	3.93	1	1.04	1.76	1.94

a) Int. : intestinal type

b) Dif. : diffuse type

Table S2. AUCs of two combined plasma biomarkers of DEK, CEA, CA19.9 and CRP in 98 GC patients and 120 healthy controls by ROC analysis.

**Combined biomarkers**	**AUC**	**SE** [a]	**Asymptotic significance** [b]
DEK+CEA	0.855	0.026	<0.0001
DEK+CA19.9	0.802	0.031	<0.0001
DEK+CRP	0.796	0.031	<0.0001
CEA+CRP	0.788	0.032	<0.0001
CEA+CA19.9	0.774	0.032	<0.0001
CA19.9+CRP	0.628	0.040	0.002

a) Under the nonparametric assumption

b) Null hypothesis: true area = 0.5 (The area under the diagonal line)

Table S3. Comparisons (*p* value) of AUCs between DEK alone and combined markers in preoperative diagnosis.

**DEK alone vs Combined**	***p* value**[a]
DEK vs DEK+CEA	0.1483
DEK vs DEK+CA19.9	0.9089

DEK vs DEK+CRP	0.9819

a) *P* value: by online calculator: “comparison of Two ROC Curves – VassarStats”
